# Discriminatory Weight of SNPs in Spike SARS-CoV-2 Variants: A Technically Rapid, Unambiguous, and Bioinformatically Validated Laboratory Approach

**DOI:** 10.3390/v14010123

**Published:** 2022-01-11

**Authors:** Nicolò Musso, Paolo Giuseppe Bonacci, Dafne Bongiorno, Stefano Stracquadanio, Dalida Angela Bivona, Concetta Ilenia Palermo, Guido Scalia, Marco Fichera, Stefania Stefani

**Affiliations:** 1 Department of Biomedical and Biotechnological Sciences (BIOMETEC), Medical Molecular Microbiology and Antibiotic Resistance Laboratory (MMAR Lab), University of Catania, 95125 Catania, Italy; nmusso@unict.it (N.M.); paolo.bonacci@unict.it (P.G.B.); d.bongiorno@unict.it (D.B.); s.stracquadanio@unict.it (S.S.); dalidabivona@gmail.com (D.A.B.); 2 4U.O.C. Laboratory Analysis Unit, A.O.U. ‘Policlinico-Vittorio Emanuele’, University of Catania, 95125 Catania, Italy; ci.palermo@policlinico.unict.it (C.I.P.); lido@unict.it (G.S.); 3 Department of Biomedical and Biotechnological Sciences, Medical Genetics, University of Catania, 95125 Catania, Italy; marco.fichera@unict.it; 4 Oasi Research Institute-IRCCS, 94018 Troina, EN, Italy

**Keywords:** COVID-19, SARS-CoV-2, COVID-19 variants, Sanger sequencing, bioinformatic validation

## Abstract

Background: The SARS-CoV-2 virus has assumed considerable importance during the COVID-19 pandemic. Its mutation rate is high, involving the spike (S) gene and thus there has been a rapid spread of new variants. Herein, we describe a rapid, easy, adaptable, and affordable workflow to uniquely identify all currently known variants through as few analyses. Our method only requires two conventional PCRs of the S gene and two Sanger sequencing reactions, and possibly another PCR/sequencing assay on a N gene portion to identify the B.1.160 lineage. Methods: We selected an S gene 1312 bp portion containing a set of SNPs useful for discriminating all variants. Mathematical, statistical, and bioinformatic analyses demonstrated that our choice allowed us to identify all variants even without looking for all related mutations, as some of them are shared by different variants (e.g., N501Y is found in the Alpha, Beta, and Gamma variants) whereas others, that are more informative, are unique (e.g., A57 distinctive to the Alpha variant). Results: A “weight” could be assigned to each mutation that may be present in the selected portion of the S gene. The method’s robustness was confirmed by analyzing 80 SARS-CoV-2-positive samples. Conclusions: Our workflow identified the variants without the need for whole-genome sequencing and with greater reliability than with commercial kits.

## 1. Introduction

In the continuous struggle among organisms, variability is the key to success. This is even more true for viruses. Mutations are random events that occur during the virus replication which can be selected or not, by the action of drugs or the immune system. As a matter of fact, viral evolution is all about mutations [1]. Mutations seem to range from 10^−8^ to 10^−6^ substitutions per nucleotide per cell infection (s/n/c) for DNA viruses and from 10^−6^ to 10^−4^ s/n/c for RNA viruses [2]. SARS-CoV-2, identified in Wuhan in 2019, is not an exception, and mutations in the spike (S) gene, responsible for the attachment and penetration of the virus into the cell, lead to new and more transmissible lineages that, in turn, are more likely to replicate and acquire other mutations. The mutations observed so far have been predominantly transitions and transversions (in particular, cytidine to uridine and guanosine to uridine) and occurred particularly due to the stress caused by therapies and vaccines [3,4]. As an example of this, the strain that quickly became predominant during the first pandemic wave (March 2020) was characterized by the D614G substitution in the S gene. This mutation was associated with enhanced entry into cells, enhanced replication in airways, and, ultimately, with higher infectiveness [5,6,7].

In the Centers for Disease Control and Prevention (CDC) guidelines for SARS-CoV-2, the term lineage (usually indicated with its PANGOLIN name) refers to a genetically closely related group of virus variants derived from a common ancestor, whilst a variant identifies a cluster of viruses having one or more mutations that differentiate it from other variants of the same virus [5,8].

Nowadays, new important SARS-CoV-2 variants descending from D614G and with increased transmissibility have been discovered. These include (i) Alpha, firstly reported in the UK; (ii) Beta, typical of South Africa; (iii) Gamma, coming from Brazil; (iv) Delta, resposible for one of the recent COVID-19 waves; (v) Kappa, first documented in India; (vi) Iota, from the United States of America; (vii) Lambda from Peru; (viii) Eta variant, representing over 20% of genomes sequenced in Nigeria and detected in more than 200 cases globally; and (ix) B.1.160, first discovered in France and widespread in Belgium, Czech Republic, Denmark, Hungary, the Netherlands, and Switzerland [9,10,11,12,13,14,15].

Announced in December 2020 and probably emerging in November 2020, the Alpha variant, the most commonly isolated variant worldwide, is characterized by the presence of the N501Y, A570D, P681H, T716I, S982A, and D1118H substitutions and deletions at positions 69/70 and 144 in the S gene [10]. The Beta variant, discovered a few days after the Alpha in samples collected between March and November 2020, also carries the N501Y mutation (phylogenetically not related to the Alpha variant) and other peculiar substitutions in the S gene—D80A, D215G, E484K, N501Y, and A701V [10]. The Gamma variant emerged in February 2020 and is associated with the E484K, K417N, N501Y, and H655Y mutations in the S gene [12].

With respect to the other less common D614G-descending lineages, apart from several mutations occurring in other genomic regions, B.1.160 is characterized by a S477N mutation in the S gene and another one in the nucleocapsid (N) gene (A376T), whereas the Eta variant shares the E484K mutation with the Beta and Gamma variants and shows new peculiar mutations in the S gene, i.e., Q52R, A67V, Q677H, and F888L [16,17].

The most recent variants, referred to as the Kappa and the Delta variants, are characterized by six mutations (E154K, L452R, E484Q, D614G, P681R, and Q1071H), and eight mutations (T19R, del156/157, R158G, L452R, T478K, P618R, D614G, and D950N) on the spike gene, respectively [18,19].

The Iota variant has six mutations on the spike gene (L5F, T95I, D253G, E484K, D614G, and A701V), whilst the Lambda variant is characterized by seven mutations on the same gene (G75V, T76I, del246/252, L452Q, F490S, D614G, and T859N) [18,19].

Last in order of arrival but certainly not least, the variant called Omicron (B.1.1.529) is characterized by more than thirty mutations on the spike gene only, many of these unique (E484A, Q493R, G496S, Q498R, N501Y, Y505H, T547K, H655Y, and N679K) while others are shared with other lineages (for example T478K with Delta and P681H with Alpha and Mu) [20].

All these mutations can be identified with our sequencing workflow [21].

In this scenario, laboratories should not just report RT-qPCR results, but they should also try to also identify the presence of already known—and even unknown—mutations, at least in the viral spike gene, especially if patients are part of an epidemiological cluster or do not respond to therapies. Several research groups have already highlighted the association between abnormal amplification curves as RT-qPCR output—suggesting an “S gene dropout”—and the presence of certain deletions in the S gene, i.e., del 69/70 [22]. This information alone is not discriminant, and sample sequencing is the only way to ascertain the presence of mutations and deletions in the gene.

Although the cost per sample of whole genome sequencing is decreasing year by year, it remains a time-consuming procedure, requiring skilled personnel and a large number of samples per run and producing large amounts of data not necessary to investigate the spreading of these variants. On the other hand, Sanger sequencing is rapid, easier to perform, and gives the required information.

In this work, we describe a rapid, easy, adaptable, and affordable workflow to detect all mutations related to the SARS-CoV-2 variants described by the WHO [11], including theDelta variant, based on two conventional PCRs and two Sanger sequencing reactions. All variants harbor both unique SNPs and shared mutations. In case of partial sequencing of the spike gene, the presence of non-unique mutations can lead to incorrect identification of the variant. In order to avoid possible errors, it is therefore necessary to sequence SARS-CoV-2 portions containing a unique and discriminating combination of SNPs that should not be randomly chosen, but rather selected based on cluster and identification. In fact, it is possible that an SNP cluster useful in discriminating certain variants may overlook others that are equally widespread in the same geographical region.

## 2. Methods and Algorithms

### 2.1. Characteristics of Samples

Eighty viral samples were delivered to our laboratory as nasopharyngeal swabs in inactivating solution or as already-extracted RNA. They came from both public and private facilities in Eastern Sicily.

The samples to be sequenced were selected using an “epidemiological and pathological filter” system in order to avoid indiscriminate sequencing. The filters included common and unique features, such as viral load no lower than the 30 threshold cycle (Ct), with conventional RT-qPCR technique; particular clinical characteristics (post-vaccination-positive patients, infection cluster, patients not responding to therapy); geographical origin (samples from patients from areas with high incidence of variants, i.e., UK, South Africa, Northern Europe); and in the case of the Alpha variant detection, the presence of S-dropouts during RT-qPCR analyses.

In case of patients from clusters such as long-term care facilities or closed groups of people, sequencing was carried out on only two samples that still presented the aforementioned common variables (see Supplementary Materials S1 for further clarification).

### 2.2. Genotyping Characterization of the Spike Gene

**Figure 1:**
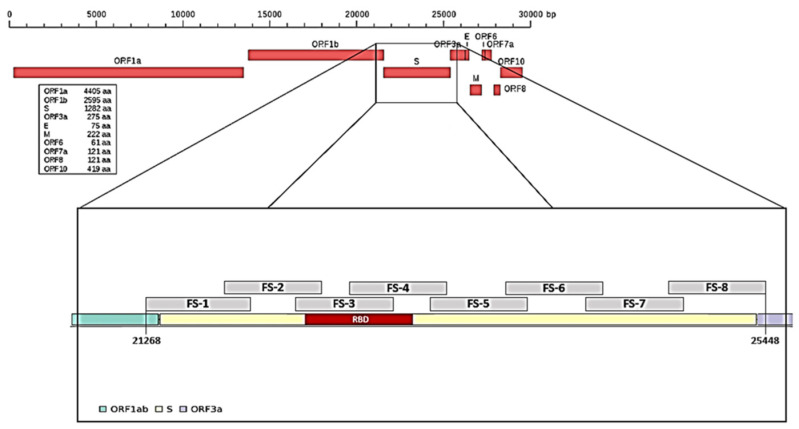
Splitting of the spike gene into eight regions (fragment spike, FS).

To characterize mutations related to the different SARS-CoV-2 variants, we arbitrarily divided the spike (S) gene into eight portions and designed a pair of primers on each portion (Table 1, Figure 1) and a pair of primers for the N gene in position 29,174–29,530, which was useful to identify the Nextstrain cluster B.1.160 lineage. We had previously performed a random reverse transcription for ten viral RNA samples; the obtained cDNA was used to test/check each pair of primers.

**Figure 2:**
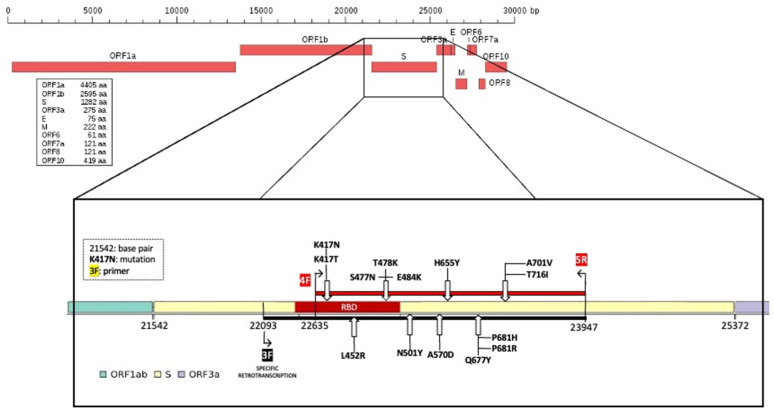
Some of the SNVs located along the spike gene detectable with the presented workflow. Black line, retro-transcribed region with specific primer (3F) and red line, amplified and sequenced “hot spot mutation fragment”.

Subsequently, for all 80 RNA samples, selective reverse transcription with the F3 primer was performed and the obtained selective cDNA was used to amplify a region of 1312 bp that we named the “Hot Spot Mutation Fragment” as it contains all mutations that allowed us to characterize the different clones (Figure 2).

### 2.3. RNA Extraction

Viral RNA from the original swabs was purified using the QIAamp^®^ Viral RNA mini-kit (Cat. No. 52904, QIAGEN, Hilden, Germany) [23]. To ensure adequate RNA extraction from samples delivered as nasopharyngeal swabs, some changes were made to the protocol provided by the manufacturer, namely, 700 µL of swab buffer was processed using only one column, instead of the original 140 µL; AVE buffer and ethanol were added in the same proportions to reach a final extraction volume of 6.3 mL, and the total volume was eluted in the same column ten times. The extracted RNA was quantified by fluorometric technique using the Qubit™ RNA HS Assay Kit (Cat. No. Q32852, Thermo Fisher, Waltham, MA, USA) according to the standard procedure. This procedure was only performed for the first nasopharyngeal swabs received, as quantification does not discriminate between human and viral RNA [24].

### 2.4. RNA Reverse Transcription and Polymerase Chain Reaction of the N and Spike Genes

RNA reverse transcription was carried out using the QuantiTect^®^ Reverse Transcription kit (Cat. No. 205311, QIAGEN, 40724 Hilden, Germany) following the manufacturer’s instructions.

As indicated by the Centers for Disease Control and Prevention (CDC) [25] to characterize the different variants, portions of the S and N genes were amplified. The spike gene was subdivided into eight fragments (fragment spike, FS, Figure 1 and Figure 2) and eight pairs of primers able to amplify each single FS were designed based on this subdivision. Primers, their positions on the reference genome, and fragment size are reported in Table 1. The primers used were designed on MN908947.3 reference sequence by using the online software Primer3 Plus.

PCRs targeting SARS-CoV-2 N and S genes were performed using Illustra^TM^ PuReTaq^TM^ Ready-To-Go^TM^ PCR Beads (Cat. No. 27955901, GE Healthcare, Chicago, IL, USA), to ensure the lowest possible levels of contaminating nucleic acids—5 µL of cDNA, 0.5% of dimethyl sulfoxide (DMSO) (Cat. No. D8418, Sigma-Aldrich-Merck KGaA, Darmstadt, Germany), and 0.5 µM of each primer. Amplifications were performed on a Biometra^®^ T3000 Thermocycler (Biorad, Segrate (MI), Italy) using the following thermal profile: initial denaturation 95 °C for 5 min, annealing at 55.5 °C for 1 min, extension at 72 °C for 1 min and denaturation at 95 °C for 1 min for 40 cycles and a final step at 72 °C for 5 min. The obtained amplicons were verified on 1.7% agarose gel in tris borate EDTA buffer (TBE) (Cat. No. B52, tris-borate-10X, Thermo Fisher Scientific, Waltham, MA, USA), stained with SYBR™ Safe DNA Gel Stain (Cat. No. S33102, Invitrogen, Thermo Fisher Scientific, Waltham, MA, USA) using a 100 bp DNA ladder (Cat. No. BR0800201, biotechrabbit GmbH, Berlin, Germany).

### 2.5. Selective RNA Reverse Transcription and Amplification of the “Hot Spot Mutation Fragment”

Selective RNA reverse transcription was carried out using the QuantiTect^®^ Reverse Transcription kit (Cat. No 205311, QIAGEN, Hilden, Germany) according to the original protocol with minor modifications, i.e., using the specific FS-3 forward primer (Table 1) at a final concentration of 1 µM. The cDNA molecule was obtained by retrotranscription and subsequently used as template during amplification of a specific stretch of the spike protein. Selective reverse transcription can be performed with all primers listed in Table 1; for FS1, the primer pair used was ORF-1.

To amplify the target region in which we had recognized the SNPs useful to identify the different variants, we performed a PCR using FS-4 forward and FS-5 reverse primers (Table 1, Figure 2), obtaining an amplicon of 1312 bp. PCRs were performed as previously described. The thermal profile used was: initial denaturation at 95 °C for 5 min, 40 cycles of annealing at 55.5 °C for 1 min, extension at 72 °C for 2 min, and denaturation 95 °C for 1 min; the final step was 72 °C for 5 min. The obtained amplicons were verified on 1.7% agarose gel, in TBE, stained with SYBR™ Safe DNA Gel Stain using a 100 bp DNA ladder.

### 2.6. PCR Amplification Products and Sequence Analysis

The amplicons obtained for the “Hot Spot Mutation Fragment” of the S gene and for the N gene were purified using the ExoSAP-IT^®^ buffer (Cat. No. 78201, Corporation, Cleveland, OH, USA) according to the manufacturer’s protocol with some modifications: the enzyme amount was increased, i.e., 3.5 μL of ExoSAP was added to 6.25 μL of PCR product. The purified amplicons were quantified using the fluorimeter Qubit dsDNA BR Assay Kit (Cat. No. 32850, Invitrogen, Carlsbad, CA, USA). Then, 15 ng of the product was sequenced in a SeqStudio Genetic Analyzer (Thermo Fisher Scientific, Waltham, MA, USA) using the Applied Biosystems BigDye terminator cycle sequencing 3.1v (Cat. No. 4337455, Thermo Fisher Scientific, Waltham, MA, USA) as previously described. The product was further purified using DyeEx^®^ 2.0 Spin Kit (Cat. No. 63206, QIAGEN, 40724 Hilden, Germany) and compared with the reference sequence “MN908947.3 severe acute respiratory syndrome coronavirus 2 isolate Wuhan-Hu-1, complete genome” (complete genome sequence release date: 18 March 2020) using the Basic Local Alignment Search Tool (BLAST, https://blast.ncbi.nlm.nih.gov/Blast.cgi, accessed on 13 December 2021) tool [26].

### 2.7. Mathematical Treatment and Calculation of Mutational Information

Through an initial screening of the Nextstrain database we selected mutations that can be considered “unique” or characteristic of specific variants to develop a mathematical treatment. An arbitrary score of 1 was assigned to each mutation and this value was divided by the number of variants sharing this mutation—if there were two variants, the mutation had a value of ½ = 0.5, and so on. In this way, a table could be created in which each mutation—with its relative score—was assigned to the virus variants (Table 2).

Starting from this table, the following term could be derived:

(1) $$\text{Mutational Percentage Explained}={\;\%}_{\text{MUT}}=\;=100\cdot \;\frac{\sum \begin{array}{c} \text{Score}\;\text{of}\;\text{mutations}\;\text{accumulated}\;\text{by}\;\text{a}\;\text{variant}\;\text{in}\\ \text{the}\;\text{gene}\;\text{stretch}\;\text{between}\;\text{FS4}\;\text{and}\;\text{FS5} \end{array}}{\sum^{}\begin{array}{c} \text{Score}\;\text{of}\;\text{mutations}\;\text{accumulated}\;\text{by}\;\text{a}\;\text{variant}\;\text{in}\\ \text{the}\;\text{whole}\;\text{pike}\;\text{gene} \end{array}}$$

In Equation (1), the score of the mutations was used in place of their amount, in order to have an idea of the weight the mutations had within the “Hot Spot Mutation Fragment”. Indeed, if the number of mutations alone was used, the analysis may have ended up including several mutations shared with other variants, resulting in erroneous information, not very useful for the purpose of univocal identification. %_MUT_ can take on values between 0 and 1, where 0 indicates lack of mutations in the sequenced gene tract, while 1 indicates that all mutations are present in the same tract.

Having introduced the term %_MUT_, the discussion can be further refined with other two terms:

(2) $$\mathrm{Degree}\;\mathrm{of}\;\mathrm{Uniqueness}=U=\frac{{\%}_{\mathrm{MUT}}\cdot \alpha }{\beta }$$

U∃∀β≠0 

This value allowed us to understand how many unique mutations were present in the gene tract sequenced. In Equation (2), α = number of mutations with value “1” present in the analyzed tract, while β = total number of mutations with value “1” present in the whole gene. It should be noted that %_MUT_ ≤ U ≤ 0. U’s maximum value can be up to %_MUT_, which would mean that the sequenced gene tract has all unique mutations in it. If U = 0, then there are no unique mutations in the same gene portion. It is important to note that if U ≠ 0, then the workflow can identify a unique mutation, and it is therefore possible to potentially identify a variant of the virus.

(3) $$\mathrm{Mutational}\;\mathrm{Density}={\;d}_{\mathrm{MUT}\;}=\frac{7650\cdot \left(\%\mathrm{MUT}\right)}{\left[2344+\left(53.06\cdot \%\mathrm{MUT}\right)\right]}\;$$

$$d_{MUT}\;\exists \forall \;\%MUT\in {\mathbb{N}}^{+}\;$$

Equation (3) was derived by considering the space in terms of base pairs covered by the workflow as the space between the first and the last detectable mutation. This is a measure of expression of the mutational information contained in the gene portion of interest (as the length in bp). This emphasizes that it was not always necessary to sequence an entire gene to obtain information that is equally satisfying. The values obtainable from Equation (3) can be between 0 and 100. If d_MUT_ = 100, then all mutations of the variant were present in that gene tract. If d_MUT_ = 0, it follows that %_MUT_ = 0, meaning no mutations were present in the aforementioned portion.

The calculation of these three parameters was performed for each variant of the COVID-19 virus in our study (Table 3).

### 2.8. Statistical Analysis

Statistical analysis was performed using Yunden’s index [27].

## 3. Results

### 3.1. Genotyping Characterization of the Spike Gene

**Figure 3:**
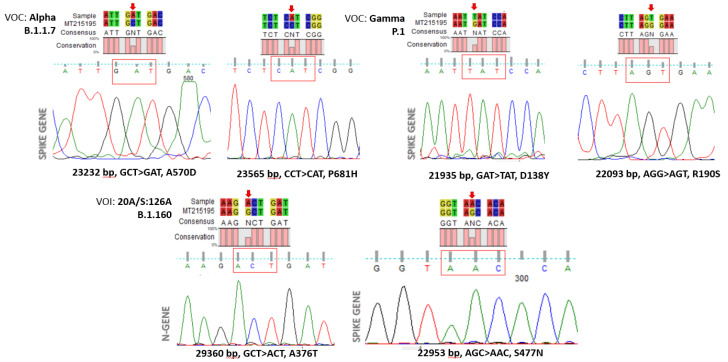
Representative chromatograms of mutations associated with specific clones.

The amplified “Hot Spot Mutation Fragment” was sequenced with the Sanger method and analyzed to find selected mutations characterizing a specific clone (Figure 3). In particular, out of 80 viral RNAs, we characterized 56 wild-type (70%), 22 samples belonging to the Alpha clone (27.5%), and two (2.5%) RNAs exhibiting mutations attributable to the Nextstrain cluster B.1.160 clone.

### 3.2. SNP Mathematical Validation

Although any test based on the sequence analysis of the shortest viral genome fragment(s) including a minimal set of SNPs carrying sufficient information to differentiate viral strains (i.e., at least one SNP and/or SNP combination unique to a viral variant) should be theoretically able to correctly characterize all variant strains under investigation, several issues may lead to incorrect results. Indeed, improper sample collection or handling, cross-contamination between patient samples, polymerase errors, and poor sequence quality, amongst other issues, may lead to an inaccurate nucleotide call and, possibly, wrong SNP evaluation. Depending on several factors, the estimation of the probability of such events is complex. However, assuming “Q” as the estimated probability of false SNP calls and considering a test aimed at differentiating between two viral strains, namely A and B, we can approximate that the viral strain A would be erroneously identified as B with a probability of “Q” when the strains differ by a single SNP and with a much smaller Q^2^ value in the case of two SNPs of difference. In the light of these considerations, it clearly emerges that the more SNPs are targeted, the more the test becomes informative, and that the test should be designed so as to balance between the two contrasting parameters, i.e., the number of the informative SNPs investigated and the length of the viral genome targeted.

In order to optimize the procedure by selecting the smallest portions of viral genome to be investigated without significant loss of test performance, we decided to analyze all possible tests targeting viral genome fragments of variable sizes for sensitivity and specificity. In this regard, we computationally built any possible subsets of discriminatory SNPs (i.e., SNPs that were unique to each variant, see Table 3) and calculated the predicted sensitivity and specificity in the identification of every specific viral variant for each of them (see Supplementary Materials for a detailed explanation and related calculations). Briefly, for any possible test based on a given subset of SNPs and for any variant “A” considered by the test, we defined:

1. Test positivity: if (a) at least one unique SNP of variant A is present and (b) no other unique SNPs characterizing other variants are detected.
2. Test negativity: if (a) no SNPs unique to variant A are identified and (b) the test is positive for variant other than A (see condition 1).
3. No call: the result does not satisfy (1) nor (2).
4. Q: the probability that an SNP is called incorrectly (i.e., it does not reflect the SNP of the viral genome tested). Q was set to a conservative value of 0.05.
5. The prevalence of each variant = 1/number of variants.

According to these rules, we estimated the number of true positive (TP), false negative (FN), false positive (FP), and true negative (TN) results for each strain and for any possible subset of unique SNPs, then we calculated the predicted sensitivity and specificity both for each specific variant and for the corresponding test.

We then filtered out any subset of unique SNPs and that enabled to discriminate all variants but one. The different tests were subsequently compared to each other taking into account sensitivity, specificity, informedness, and length of the viral genome that would be targeted by the test (See Supplementary Materials S2 for further clarification).

### 3.3. Uniqueness of Mutations in the “Hot Spot Mutation Fragment”

Some of the mutations considered in our work and reported in the literature can be defined as “unique”. In fact, these mutations were characteristic of some specific variants (e.g., the A570D mutation for the Alpha variant) and, consequently, these mutations were more important in the process of recognizing the SARS-CoV-2 variants. The mathematical model developed, allowed us to choose unambiguously the S-gene fragment containing the representative mutation characteristic of the VOC.

Focusing on this “Hot Spot Mutation Fragment” (Table 4), we observed that the Alpha variant has a %_MUT_ of about half (45.43%), and this was because the portion we sequenced covered a number of Alpha mutations corresponding to the number of mutations in the non-sequenced S gene region, although one of the mutations we detected with our method had a more major impact than del 69–70, because the %_MUT_ value was not exactly 50. Consequently, U was half of %_MUT_, since only half of the unique mutations present in the spike gene for this variant and described in the literature fell within the sequenced tract. Similar results were obtained for Beta and Kappa variants.

As for the characterization of the Gamma variant, our workflow allowed us to sequence two out of the four unique variants considered, which explains why the aforementioned indicators had values that are not very high, but still sufficient to distinguish this variant from the others (61.24%_MUT_).

The gene tract considered in the study allowed us to unambiguously identify the Eta variant (U = 7.35), presenting a reliable value of %_MUT_ (29.41%) and an excellent value for d_MUT_ (57.62%). The same results were assumed for Delta and Iota variants.

Finally, with regard to the B.1.160 clone, the two unique mutations can be identified by sequencing both the N gene and the S gene (in this case all the indicators were equal to 100).

### 3.4. Bioinformatic Validation

Among all possible tests based on 176 non-redundant subsets of unique mutations, 49 were able to discriminate all variants, while 71 allowed the identification of all but one variant. Assays based on these subsets were then selected as possible candidate tests and compared to each other in terms of sensitivity, specificity, and length of viral genome fragments. Clearly, the sensitivity and specificity of the tests correlated with the number of SNPs investigated and their distribution across variants. Indeed, higher sensitivities and specificities were obtained by tests targeting the many SNPs homogenously distributed among variants (maximum: 0.98, number of unique SNPs: 16, mean: 1.77, S.D.: 0.4; minimum: 0.83, number of unique SNPs: 16, mean: 1.77, S.D.: 1.85).

To balance our evaluation by giving an equal weight to sensitivity and specificity and to summarize the performance of the tests, we compared our results using the Youden’s index (informedness or sensitivity + specificity −1). The informedness ranged from a maximum of 0.93 to a minimum of 0.73, which did not strictly correlate either with the length of the viral genome investigated or with the number of SNPs targeted (3043 nt, 16 SNPs; 3296 nt, 16 SNPs, respectively).

Aiming to select a test relying on the smallest number of fragments to be amplified without a significant loss of informedness, we ordered the tests based on the length of the viral genome to be sequenced (see Table 3 and Appendix A Table A1).

As shown in Table 3, the tests based on shorter fragments did not significantly differ in terms of maximum informedness, suggesting that more parsimonious tests could be reliably used to discriminate viral variants. According to these observations, the subset including SNPs from R158G to T716I, sizing 1603 bp, would have been an excellent target for the test. From a practical point of view, however, the efficiency of Sanger sequencing of such a viral genome fragment would have been impaired by the amplicon length. To investigate whether targeting shorter fragments would still result in a test with acceptable sensitivity and specificity, we further analyzed the performance of tests that did not capture the entire spectrum of variants. Considering several parameters, including estimated informedness and amplicon lengths, we observed that the subset comprising the SNPs K417N, N439K, Y453F, S477N, T478K, E484K, N501Y, A570D, H655Y, Q677H, P681H, I692V, A701V, and716I, would lead to a test providing an acceptable compromise between informedness (0.76) and minimal amplicon length (896 bp). It should be noted that, owing to the lack of the A222V mutation, the Nextstrain Cluster 20A.EU.1 variant was overlooked by that test, causing a drop to 0.84 in the estimated overall sensitivity. Nevertheless, we selected this latter subset as the best candidate to target, aiming at speed and therefore at the efficiency of the entire workflow. Indeed, it should be evidenced that the Nextstrain Cluster 20A.EU.1 represents a minor variant and its prevalence was probably overestimated in our model (leading to an underestimation of the overall sensitivity). On the other hand, the length of the required amplicon was halved and the time required to complete the PCR amplification was reduced by 1/3 compared to amplifying the fragment that would enable the identification of all variants.

In any case, the A222V mutation, the only unique mutation for the Nextstrain Cluster 20A.EU.1” variant, can be sequenced using three primer combinations constantly provided in our work (FS-1F/FS-2R; FS-2F/FS-3R; and FS-3F/FS-4R). From a practical point of view, however, the amplification with these primer combinations could be carried out in parallel with the reactions for our “Hot Spot Mutation Fragment”, as the annealing temperature of the aforementioned primer pairs coincides with the temperature set for our fragment. The exclusion of the R215D mutation, on the other hand, is compensated by other mutations characterizing the South African variant which were sequenced with our selected workflow (FS-4F/FS-5R).

## 4. Discussion

Recent genotyping analysis demonstrated that genes that encode the spike protein, other nucleoprotein, and RNA polymerase undergo very frequent mutations. In particular, single high-frequency SNPs in SARS-CoV-2 were found on the spike glycoprotein (D614G, 23364 A > G), as well as in the protein encoding for the nucleocapsid (R203K, R202R, and G204R) [28].

The combination of SNPs—and the consequent formation of genetic clusters—is crucial in SARS-CoV-2 variant identification, this being a key point in the management of the pandemic, considering their clinical and epidemiological implications. In fact, variants change the ability of the virus to bind the human receptor ACE2 [29] thus making it more infective, as in the case of the N501Y mutation.

This genomics investigation aimed to identify a new, rapid, unambiguous, and bioinformatically validated sequencing method to detect the more frequent VOI without performing NGS of all viral genomes. This method proved to be simple, univocal, hands-on, and cost-effective.

The chosen portion of the SARS-CoV-2 S gene contains a set of SNPs which are useful to discriminate many variants with only few reactions. Our test, validated on 80 viral genomes was able to correctly detect major VOC, such as Alfa, Beta, and Gamma, without errors.

Furthermore, our method, which involves the sequencing of a specific portion of spike gene, allows the identification and sequencing of the most recent Omicron variant, not contemplated in the tables as it was announced at the end of the work.

Our computational model predicts that our assay will remain sufficiently robust to laboratory issues that may lead to incorrect base-calling and inaccurate variant-determination.

## 5. Conclusions

The use of a mathematical tool to unambiguously predict mutation strength, weight, and SNP combinations allowed us to identity precisely variants with only a few laboratory reactions, requiring fewer trained lab technicians and with the advantage of being easier to use than the more cumbersome NGS. The tool identified 7616 combinations for the unique discrimination of the to-date-known variants.

## Figures and Tables

**Table 1:** Oligonucleotide sequence for spike gene fragments (FS) and N gene. F, forward primers; R, reverse primers.

Gene/Primer Name		Primer Sequences 5′-3′	Position in Reference Genome	Amplicon Size (bp)
ORF-1	F	CCGTGGCTATAAAGATAACAGAACATT	21,116	-
Spike				
FS-1	F	GAACAAATAGATGGTTATGTCATGC	21,268	666
	R	ACTAGGTAAAAACCCACAAA	21,934	
FS-2	F	CACGTGGTGTTTATTACCCTGA	21,621	803
	R	ACATGCAACTTTAGGAAGTGAC	22,424	
FS-3	F	GGAATTTGTGTTTAAGAATATTGATGG	22,093	843
	R	TTAGATAGTCCGGCCATCGT	22,936	
FS-4	F	CCGCATCATTTTCCACTTTT	22,635	825
	R	CACGTCCGACAAATTATCCCC	23,460	
FS-5	F	TTTGGTTAAAAACAAATGTGTCAA	23,119	828
	R	AGGTAGTTTTGGTTCGTTCT	23,947	
FS-6	F	GGTGCAGAAAATTCAGTTGC	23,621	809
	R	AAAACCACGTTAAAGTTCACAA	24,430	
FS-7	F	ACTGTTTTGCCACCTTTGCT	24,098	839
	R	ACTAGGAAACGTTGGACTTAA	24,937	
FS-8	F	TCAGAGTGTGTACTTGGACAATCA	24,611	818
	R	AGTCTAAAACAAGCGCGAT	25,429	
N1	F	TTCTTCGGAATGTCGCGCA	29,174	356
	R	TTTGCAAAAGCGAAAAGGCA	29,530	

**Table 2:** Mutational score of the most circulating variants in Europe.

	Variants of Concern				Variants of Interest						Variants of Concern				Variants of Interest				
WHO Label	α	β	γ	δ	κ	ι	λ	η	B.1.160		α	β	γ	δ	κ	ι	λ	η	B.1.160
L5F						1				L452R				0.5	0.5				
T19R				1						Y453F									
Del 69-70	1									S477N									1
Q52R								1		T478K				1					
A67V								1		E484Q					1				
G75V							1			E484K		0.25	0.25			0.25		0.25	
T76I							1			F490S							1		
D80A		1								N501Y	0.33	0.33	0.33						
T95I						1				A570D	1								
Del 144	1									H655Y			1						
E154K					1					Q677H								1	
del156_157				1						P681R				0.5	0.5				
R158G				1						P681H	1								
D215G		1								I692V									
A222V										A701V		0.5				0.5			
del246_252							1			T716I	1								
D253N										T859N							1		
D253G						1				F888L								1	
K417T			1							D950N				1					
K417N		1								S982A	1								
N439K										Q1071H					1				
L452Q							1			D1118H	1								

**Table 3:** %_MUT_, U, and d_MUT_ values refer to the most common circulating variants in Europe calculated for amplicons obtainable from all possible primer combinations.

	%_MUT_							U							d_MUT_						
	F1-R2	F2-R3	F3-R4	F4-R5	F5-R6	F6-R7	F7-R8	F1-R2	F2-R3	F3-R4	F4-R5	F5-R6	F6-R7	F7-R8	F1-R2	F2-R3	F3-R4	F4-R5	F5-R6	F6-R7	F7-R8
Alpha	27.29	13.64	18.14	45.43	40.93	40.93	27.29	7.80	1.95	2.59	19.47	17.54	17.54	7.80	55.05	34.02	41.98	73.10	69.34	69.34	55.05
Beta	49.02	73.53	63.24	50.98	12.25	0.00	0.00	32.68	73.53	42.16	16.99	0.00	0.00	0.00	75.83	90.07	84.88	77.24	31.31	0.00	0.00
Gamma	0.00	38.76	61.24	61.24	38.76	0.00	0.00	0.00	19.38	30.62	30.62	19.38	0.00	0.00	0.00	67.38	83.76	83.76	67.38	0.00	0.00
Delta	50.00	41.67	25.00	33.33	8.33	16.67	16.67	30.00	16.67	5.00	6.67	0.00	3.33	3.33	76.55	69.98	52.10	62.00	22.88	39.49	39.49
Kappa	25.00	37.50	37.50	50.00	12.50	25.00	25.00	8.33	12.50	12.50	16.67	0.00	8.33	8.33	52.10	66.20	66.20	76.55	31.80	52.10	52.10
Iota	53.33	53.33	33.33	20.00	13.33	0.00	0.00	35.56	35.56	11.11	0.00	0.00	0.00	0.00	78.86	78.86	62.00	44.93	33.43	0.00	0.00
Lambda	33.33	66.67	50.00	33.33	16.67	16.67	16.67	11.11	44.44	25.00	11.11	0.00	2.78	0.00	62.00	86.72	76.55	62.00	39.49	39.49	39.49
Eta	47.06	47.06	5.88	29.41	47.06	23.53	23.53	23.53	23.53	0.00	7.35	11.76	5.88	5.88	74.37	74.37	16.94	57.62	74.37	50.10	50.10
B.1.160	0.00	100.00	100.00	100.00	0.00	0.00	0.00	0.00	100.00	100.00	100.00	0.00	0.00	0.00	0.00	100.00	100.00	100.00	0.00	0.00	0.00

**Table 4:** Values of %_MUT_, U, and d_MUT_ sequencing the gene portion between FS-4 and FS-5.

	%_MUT_	U	d_MUT_
Alpha	45.43	19.47	73.10
Beta	50.98	16.99	77.24
Gamma	61.24	30.62	83.76
Delta	33.33	6.67	62.00
Kappa	50.00	16.67	76.55
Iota	20.00	0	44.93
Lambda	33.33	11.11	62.00
Eta	29.41	7.35	57.62
B.1.160	100	100	100

## Data Availability

Not applicable.

## Supplementary Materials

The following supporting information can be downloaded at: https://www.mdpi.com/article/10.3390/v14010123/s1, Supplementary information S1: Modifications to the sequencing protocols; Supplementary information S2: Sensitivity and Specificity estimation of tests based on different mutation combinations.

## Appendix A

**Table A1:** Combinations of bioinformatically validated SNPs, with correlated information derived from computational analysis.

**SNPs Tested**	**Sensitivity**	**Specificity**	**Information**	**Minimal Length of the Spike Gene Tested**	**Number of Unique Mutations Tested**
-del144-Q52R-D215G-A222V-K417N-N439K-Y453F-S477N	0.93	0.95	0.89	1285	8
-D215G-A222V-K417N-N439K-Y453F-S477N-A570D-H655Y-Q677H-P681H	0.94	0.95	0.89	1398	10
-D215G-A222V-K417N-N439K-Y453F-S477N-A570D-H655Y-Q677H-P681H-I692V-A701V-T716I	0.96	0.95	0.91	1503	13
-del144-Q52R-D215G-A222V-K417N-N439K-Y453F-S477N-A570D	0.94	0.95	0.89	1564	9
-del144-Q52R-D215G-A222V-K417N-N439K-Y453F-S477N-A570D-H655Y	0.94	0.95	0.89	1820	10
-del144-Q52R-D215G-A222V-K417N-N439K-Y453F-S477N-A570D-H655Y-Q677H-P681H	0.95	0.95	0.90	1897	12
-del144-Q52R-D215G-A222V-K417N-N439K-Y453F-S477N-A570D-H655Y-Q677H-P681H-I692V-A701V-T716I	0.97	0.95	0.92	2002	15
-D215G-A222V-K417N-N439K-Y453F-S477N-A570D-H655Y-Q677H-P681H-I692V-A701V-T716I-F888L	0.97	0.95	0.92	2018	14
-D215G-A222V-K417N-N439K-Y453F-S477N-A570D-H655Y-Q677H-P681H-I692V-A701V-T716I-F888L -S982A	0.97	0.95	0.92	2300	15
-del144-Q52R-D215G-A222V-K417N-N439K-Y453F-S477N-A570D-H655Y-Q677H-P681H-I692V-A701V-T716I-F888L	0.97	0.94	0.91	2517	16
