# Supplementary material for: Discriminatory Weight of SNPs in Spike SARS-CoV-2 Variants: A Technically Rapid, Unambiguous, and Bioinformatically Validated Laboratory Approach
Source: Viruses. 2022 Jan 11;14(1):123. doi: 10.3390/v14010123 (PMC8781109; doi:10.3390/v14010123)
Supplement: Supplementary file 1 [file viruses-14-00123-s001.zip › viruses-1529789-supplementary.pdf]

Supplementary Information S1

Despite low Threshold Cycles (CTs <30) being a prerequisite for sequencing in our work, it was neglected in cases where samples presented other important characteristics, such as their origin from areas with high epidemiological incidence. In cases like these, samples with CTs well above 30 were also sequenced, making slight modifications to the sequencing protocol.

RNA precipitation

Before proceeding with selective reverse transcription, samples underwent a treatment aimed to precipitate and concentrate the RNA. This was carried out by treating the samples with 3M Sodium Acetate and Absolute Ethanol, -20 °C overnight. Two further washes were subsequently carried out with 75% ethanol, after which the precipitated RNA was resuspended in Nuclease Free Water.

Nested-PCR

At the end of PCR, two Nested-PCRs were performed on these samples, amplifying FS-4 and FS-5 individually, thus managing to obtain amplicons that can be sequenced even in the presence of particularly high CTs.

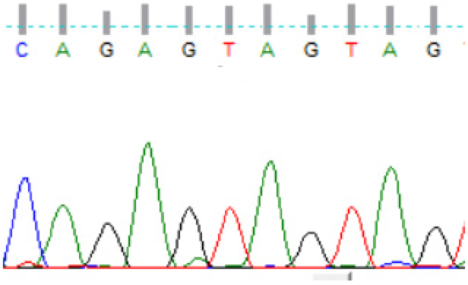

Figure S1. Electropherogram of a sequence subjected to RNA precipitation and Nested-PCR from sample with 36.79CT in qRT-PCR for the Spike gene.

Supplementary Information S2

Sensitivity and Specificity estimation of tests based on different mutation combinations

In order to estimate the sensitivity and specificity for each SARS-CoV-2 variant when considering tests based on different possible combinations of targeted mutations, we have taken into consideration only the mutations which are unique to each variant.

To this purpose we defined

a) ***S*** as the set of the N=8 SARS-CoV-2 variants

$$S = \{A, \dots, i, \dots . H\} \qquad i = A, B, \dots, H \quad i \neq Ref$$

b) ***X*** as the set of unique mutations

$\boldsymbol{X} = \{M_1, M_2, .., M_{20}\}$ where M_1, M_2, …, M_{20} are unique mutations

c) The variant A, B, …H in relation to the unique mutations:

$$\begin{cases} A, \dots, i, \dots, H \subset X \; : \; i \cap j = \emptyset \;\; \forall i \neq j \\ A \cup B \cup \dots \cup H = X \end{cases}$$

d) $\boldsymbol{\mathcal{P}}(\boldsymbol{X})$ the power set of the set ***X***

$$\boldsymbol{\mathcal{P}}(\boldsymbol{X}) = \left\{\{\boldsymbol{M_1}\}, \dots, \{\boldsymbol{M_n}\}, \dots, \; \{\boldsymbol{M_i}, \boldsymbol{M_j}\}, \dots, \{M_1, M_2, .., M_{20}\}\right\}$$

e) U_i the number of unique mutations of a variant of an element Y of $\mathcal{P}(\boldsymbol{X})$

$$U_i = n(i \cap Y) \qquad \text{i=A, B, …, H}$$

f) U_T the total number of unique mutations of Y

$$U_T = n(Y)$$

g) Q: the probability that a SNP is called incorrectly (i.e., it does not reflect the SNP of the viral genome tested). Q was set to a conservative value of 0.05

h) P the probability that the SNPs is called correctly P=1-Q

i) The prevalence of each variant = 1/ number of variants.

We then computationally built the elements of $\mathcal{P}(X)$ and estimated for each of them the sensitivity and specificity of each test that would be based on a specific element (i.e. combinations of unique mutations).

We subsequently filtered out any result (i.e. subset) that did not allow at least the discrimination of all but one variants. Eventually, the different tests were compared each other taking into account the sensitivity, specificity and length of the viral genome that would be targeted by the test.

Estimation of TP, FN, TN and FP

We calculate at first TP, FN, TN and FP related to variant A in the case of 4 variants (A, B, C, D) and the generalize the final equations.

1) **True Positive (TP)**

Test for strain A gives A (i.e. true positive result) if:

- At least one unique mutation of A remains true (i.e. 1):

$$(1 - Q^{UA}) \quad \text{i.e} \quad \text{1-p (all are false)} \qquad \text{eq. 1}$$

and

- All mutations of the remaining strains are true (i.e. 0):

$$(P)^{UB} * (P)^{UC} * (P)^{UD} = (P)^{UT-UA} \qquad \text{eq. 2}$$

Finally,

$$p(TP|A) = (1 - Q^{UA}) * (P)^{UT-UA} \qquad \text{eq.3}$$

- *Which may be generalized to*

$$\boldsymbol{p(TP|i) = (1 - Q^{Ui}) * (P)^{UT-Ui} \qquad i \in S} \qquad \text{eq. 4}$$

For the reference strain

$$\boldsymbol{p(TP|ref) = (P)^{UT}} \qquad \text{eq. 5}$$

2) **False Negative (FN)**

Test for strain A gives B or C or D (i.e. false negative result) if:

1) No UA remains true (i.e. remains 1) i.e. all UA are false

$$Q^{UA} \qquad \text{eq.6}$$

And 2)

2) At least 1 UB becomes (i.e. becomes 1) false and all UC and UD remain true

$$(1-(P)^{UB}) * (P)^{UC} * (P)^{UD} \qquad \text{eq. 7}$$

which can be written:

a) $$(P)^{UT-UA-UB} - (P)^{UT-UA} \qquad \text{eq. 8}$$

Or b)

1 UC becomes false and all UB and UD remain true

b) $$\boldsymbol{P}^{UT-UA-UC} - \boldsymbol{P}^{UT-UA} \qquad \text{eq. 9}$$

or c)

at least 1 UD becomes false and all UB and UC remain true

c) $$P^{UT-UA-UD} - P^{UT-UA} \qquad \text{eq. 10}$$

by adding eq.8, eq.9, and eq.10

$$P^{UT-UA-UB} - P^{UT-UA} + P^{UT-UA-UC} - P^{UT-UA} + P^{UT-UA-UD} - P^{UT-UA} \qquad \text{eq. 11}$$

which can be written:

$$P^{UT-UA-UB} + P^{-UT-UA-UC} + P^{UT-UA-UD} - 3P^{UT-UA} \qquad \text{eq.12}$$

We also have to add the term related to the reference genome (i.e. the probability that the strain A is call as reference by the test) to that equation:

$$P^{UT-UA} \qquad \text{eq. 13}$$

Finally, combining eq.1, eq.12 and eq.13 together:

$$p(FN|A) = Q^{UA} * [P^{UT-UA-UB} + P^{UT-UA-UC} + P^{UT-UA-UD} - 2P^{UT-UA}] \qquad \text{eq. 14}$$

- *Which may be generalized to*

$$\boldsymbol{Q^{Ui}P^{UT-Ui}\left[\sum_{j\neq i} P^{-Uj} - (N-2)\right]} \qquad \text{eq. 15}$$

Which becomes for the reference strain:

$$p(FN|ref) = P^{UT-UA} - P^{UT} + P^{UT-UB} - P^{UT} + P^{UT-UC} - P^{UT} + P^{UT-UD} - P^{UT} \qquad \text{eq. 16}$$

- *Which may be generalized to*

$$\boldsymbol{p(FN|ref) = P^{UT}\left[\sum_{i} P^{-Ui} - N\right]}$$

3) No Call

The test does not give interpretable results (i.e neither true positive nor false negative) in the remaining cases

$$\boldsymbol{p - (Nocall|i) = 1 - TP(i) - FN(i)} \qquad \text{eq.17}$$

4) False positive

Referring to variant A, false positive may arise if Strain B or Strain C or Strain D or reference strain are called as strain A by the test

- Let's calculate this probability for strain B (i.e. Strain B results in Strain A)
  There are 3 conditions that must be met

  1) No UB remains true (i.e. 1 remains 1)

$$Q^{UB} \qquad \text{eq.18}$$

  2) At least 1 UA becomes false (i.e. 0 becomes 1)

$$1 - P^{UA}$$ eq.19

3) Both UC and UD remain true (i.e. 1 remains 1)

$$P^{UC} P^{UD}$$ eq.20

By combining eq.18, eq.19, and eq.20 together

$$Q^{UB} * (1 - P^{UA}) * P^{UC} * P^{UD}$$ eq. 21

Which may be written as:

$$(1 - P^{UA}) * Q^{UB} * P^{UT-UA-UB}$$ eq. 22

- Considering that false positives may also arise from other strains:

$$(1 - P^{UA}) * [Q^{UB} * P^{UT-UA-UB} + Q^{UC} * P^{UT-UA-UC} + Q^{UD} * P^{UT-UA-UD}]$$ eq.23

As well as from the reference strain:

$$(1 - P^{UA}) * P^{UT-UA}$$ eq.24

$$p(FP|A) = (1 - P^{UA}) * [Q^{UB} * P^{UT-UA-UB} + Q^{UC} * P^{UT-UA-UC} + Q^{UD} * P^{UT-UA-UD} + P^{UT-UA}]$$ eq.25

Which can be written

$$p(FP|A) = (1 - P^{UA})P^{UT-UA}[Q^{UB} P^{-UB} + Q^{UC} P^{-UC} + Q^{UD} P^{-UD} + 1]$$ eq.26

Which may be generalized to

$$\boldsymbol{p(FP|i) = (1 - P^{U_i})P^{UT-Ui}\left(\sum_{j \neq i} Q^{Uj} P^{-Uj} + 1\right)}$$ eq. 27

The overall probability for the variant strains to be called as reference will be:

$$p(FP|A,B,C,D) = Q^{UA} * P^{UT-UA} + Q^{UB} * P^{UT-UB} + Q^{UC} * P^{UT-UC} + Q^{UD} * P^{UT-UD}$$ eq.28

Which may be generalized to

$$\boldsymbol{p(FP|A,\dots,i,\dots,H) = \sum_i Q^{U_i}\, P^{UT-U_i}}$$ eq. 29

5) True Negative

Referring to variant A, true negative is variant different than variant A that are correctly called non-A by the test.

$$p(TN|A) = 1-(1-P^{UA})P^{UT-UA}[Q^{UB}\,P^{-UB} + Q^{UC}\,P^{-UC} + Q^{UD}\,P^{-UD} + 1]$$ eq. 30

Which may be generalized to

$$\boldsymbol{p(TN|i) = 1-(1-P^{U_i})P^{UT-Ui}\left(\sum_{j\neq i} Q^{Uj}P^{-Uj} + 1\right)}$$ eq.31

The overall probability for the variant strains different from reference to be correctly called non-reference by the test will be:

$$p(TN|A,B,C,D) = 1-(Q^{UA}P^{UT-UA} + Q^{UB}P^{UT-UB} + Q^{UC}P^{UT-UC} + Q^{UD}P^{UT-UD})$$ eq.32

Which may be generalized to

$$\boldsymbol{p(TN|A,\dots,i,\dots,H) = 1-\sum_i Q^{U_i}\, P^{UT-U_i}}$$ eq.33

- **Note:** the calculations were performed considering all the strains as having the same prevalence.
